# A qualitative socioecological exploration of community health promoters’ barriers and facilitators in cardiovascular disease prevention in Kenya

**DOI:** 10.3389/fpubh.2026.1790036

**Published:** 2026-04-24

**Authors:** Emily N. Muchina, Maureen Akolo, Rose Maina

**Affiliations:** School of Nursing and Midwifery, Aga Khan University, Nairobi, Kenya

**Keywords:** barriers and facilitators, cardiovascular disease prevention, community health promoters, qualitative study, socioecological model

## Abstract

**Background:**

Cardiovascular diseases (CVDs) remain a growing public health challenge in low- and middle-income countries, where there is an upsurge of risk factors. Community health promoters (CHPs) have the potential to play a crucial role in CVD prevention and care to underserved communities in Kenya. However, there are limited studies on their experiences, which are likely shaped by multilevel barriers and facilitators across the individual CHP, the community, relationships with primary health systems, and societal-level policies and structures. This study explored the barriers and facilitators shaping CHPs’ experiences in mitigating CVD in Kenya.

**Methods:**

A qualitative descriptive study was conducted in the Lari sub-county of Kiambu. Data were collected using semi-structured interview guides with 15 purposively selected CHPs and 6 key informants, including supervisors and health facility incharges, guided by the principle of data saturation. Qualitative content analysis was employed, guided by a socio-ecological framework.

**Results:**

Findings revealed a complex interplay of barriers and facilitators across four themes: individual, relationship, community, and society. CHPs had a limited understanding of CVDs, revealing inadequate CVD training. Commitment and remuneration were enabling; however, CHPs faced limited financial support and a heavy workload. Community acceptance and leadership support enhanced outreaches, whereas poverty and resistance, rooted in cultural and religious beliefs, hindered uptake. Despite supervision and teamwork within the health system, staffing gaps and medication shortages persist. At the societal level, role recognition and functional referral structures legitimised the CHP efforts, though inadequate resources undermined sustainability.

**Conclusion:**

CHP’s commitment, community acceptance, supportive leadership, supervision, role recognition, and functional referral structures enhanced CHP’s motivation and service. However, limited CVD knowledge, inadequate training, financial difficulties, community poverty and resistance, staffing, medication and resource shortages impede their capacity to reduce CVD. Strengthening their role requires multilevel interventions, including structured training, adequate resourcing, and leveraging community trust, to sustain CVD prevention in Kenya and similar low-resource contexts.

## Introduction

Cardiovascular diseases (CVDs) have emerged as the leading cause of morbidity and mortality in the world, accounting for nearly a third of all deaths worldwide (1). More than three quarters of these deaths take place in low- and middle-income countries (LMICs) (1), where a considerable burden of CVD fatalities is found outside of healthcare settings (2). Yet, CVDs are preventable by reducing both modifiable and non-modifiable risk factors. The burden of CVD is high, which calls for intense preventive interventions by focusing on the reduction of CVD risk factors. While high-income countries have witnessed declining CVD mortality rates due to improved prevention and treatment strategies (3, 4) LMICs, particularly sub-Saharan Africa, continue to experience a growing burden of CVD and its risk factors (1). An epidemiological change in Africa has been characterised by a rising burden of non-communicable diseases over the last three decades, despite the sustained burden of infectious diseases (5). Notably, CVD is a leading contributor to the burden due to a heightened and unimpeded CVD risk factor state, particularly hypertension (6, 7). In the region, limited access to health services, gaps in early detection, and poor community awareness aggravate CVD challenges (8). Kenya mirrors these global trends, with communities facing widespread exposure to CVD risk factors including hypertension, diabetes, obesity, unhealthy diet, physical inactivity and use of tobacco while still struggling with infectious diseases, creating a double burden of disease with an already constrained health system (9, 10). Similarly, the Kiambu region in Kenya has experienced a rise in CVD burden and its risk factors, especially hypertension (11, 12). This is attributable to urbanisation and lifestyle changes. Yet community-based efforts in the prevention of CVD are low (13).

To overcome the CVD burden, LMICs must prioritise cost-effective, community-based CVD prevention (14). The use of community-based CVD prevention interventions to overcome the mounting burden of CVD and early loss of life is crucial and timely. Engaging community health promoters (CHPs), otherwise referred to as community health workers, community health volunteers, community health extension workers, village or lay health workers, has been acknowledged as an effective strategy to reach marginalised populations, advance worldwide health coverage, and reduce health inequities (15, 16). CHPs are essential in Kenya’s context of a rising CVD burden because they are embedded within communities since 2006 and have taken on the role to provide screening, lifestyle counselling, referrals, and follow-up care at the household level in the prevention of CVD (17). This is in addition to their traditional role of prevention and care for maternal, child, and communicable diseases. CHPs thus serve as frontline workers, bridging the gap between households and primary care; however, their role in CVD prevention is hindered by multilevel challenges that remain insufficiently studied. Existing research on CHPs in Africa focuses primarily on maternal, child and infectious diseases, with limited emphasis on their CVD role. Regional studies in Uganda, Rwanda, and South Africa indicate that CHP-led CVD interventions are feasible and acceptable; however, they are constrained by training gaps, inadequate resources, and poor system support (18–20). CHPs’ experiences in CVD prevention in rural Kenya are underexplored, where health system constraints and community dynamics shape outcomes (10). In 2023, the Kenyan government enhanced community health services to tackle non-communicable diseases, including CVDs, by introducing standardised CHP kits with screening tools such as blood pressure monitors and glucometers (21). Additionally, they committed to paying stipends and health insurance for CHPs. Yet evidence indicates ongoing structural and operational barriers hinder CHP effectiveness. Kagwanja et al. (22) noted fragmented hypertension care due to financial issues, supply chain problems, insufficient community and provider awareness, and poor coordination, despite motivational factors and existing health infrastructure.

Evidence on how factors at different levels interact to shape CHPs’ role in CVD prevention is lacking, despite existing policies in Kenya to advance community-based prevention (23, 24). This hampers the implementation of tailored, context-based strategies in primary healthcare. This study employed a qualitative inquiry to explore the multilevel barriers and facilitators shaping the implementation of CVD prevention by CHPs in Kenya. The aim was to guide policy, practise, and research to strengthen CVD prevention in Kenya and other LMICs.

## Materials and methods

### Study design

This study utilised a qualitative descriptive design, appropriate for exploring the realities of CHPs in mitigating CVDs. This helped a straightforward exploration of participants’ views expressed in their own words (25). Guided by a constructionist view of reality Adom et al. (26), the design recognised the diversity of experiences among CHPs and produced findings in a language close to that used in practise (27).

### Setting and sample

The setting was Kiambu County, Lari Subcounty, a largely rural agricultural area, located in the western part of the county. It suffers the effects of urbanisation due to its proximity to Nairobi, the capital City in Kenya. The Subcounty has five geographical wards, namely Kamburu, Kinale, Kirenga, Nyanduma, and Kijabe, from which link or referral primary health care facilities, including level 2 and 3, are located. Each health facility is headed by an in-charge who is either a nurse or a clinician. Every ward has a community health assistant (CHA) who supervises and coordinates the CHPs’ health activities. The lowest health care level is level one, which is a collection of households where about 233 CHPs are attached, each is responsible for 100 households (24).

Twenty-one participants were purposively recruited with the assistance of the community health focal person responsible for the community health strategy in Lari Subcounty. He was instrumental in identifying eligible CHPs across the five different wards who were actively engaged in community health roles and had more than 1 year of experience. The CHAs supervising them were included as participants for their insight into CHP roles. Additionally, link facility in-charges who were present at the time of data collection were recruited as participants. Key informants’ views were crucial in understanding how CHPs’ experiences were shaped by various factors. Fifteen CHPs and six key informants, including CHAs and link facility incharges participated in the study, guided by the principle of saturation. Participants were coded by numbering them sequentially based on interview order as (CHPs P1-P15) and Key informants (KI1-KI6).

### Data collection

Data were collected from January to February 2025 through face-to-face interviews using semi-structured and open-ended interview guides in either Kiswahili or English. Probes were designed to elicit more detailed information from participants. The interview guides were first pretested with 3 CHPs and a key informant to ensure clarity and appropriateness of context (28). No amendments were made, except for the addition of probes during the interview, and the participants were not included in the actual study.

The first author first contacted the identified participants and arranged meetings at their respective link health facilities. The study purpose was explained in detail, and their written consent was sought before data collection. Before the interview began, introductions were made to build rapport with each participant. Interviews were audio-recorded in a private room within the referral health facilities to minimise noise and distractions. Interviews, averaging 33 min, were conducted in Kiswahili or English, based on participants’ preferences, until data saturation was reached, as indicated by the repetition of responses and the absence of new information during analysis (25). Field notes captured nonverbal cues, along with researchers’ reflections on the data collection process.

### Theoretical framework

**Figure 1:**
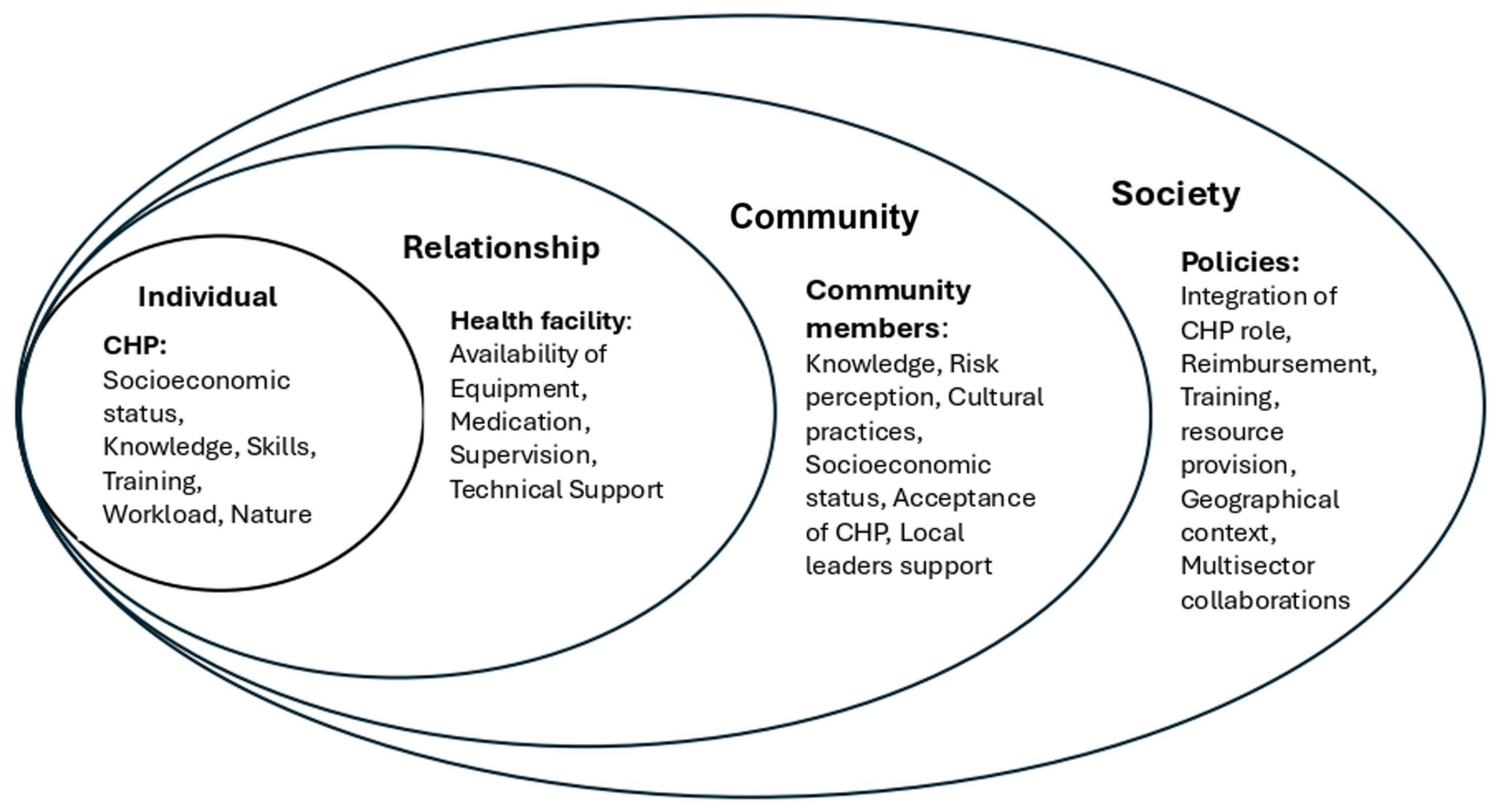
The social ecological model: a framework for prevention. Adapted from Heise (31) and modified with variables influencing CHPs in the implementation of CVD prevention.

The socioecological model guided this study, a framework initially developed by the psychologist Urie Bronfenbrenner in the 1970s (29). It was later adapted for public health by McLeroy and colleagues in 1988, as a practical model for assessing health behaviours and community interventions (30). This study was operationalised through Lori Heise’s four-level socioecological framework Heise (31), which encompasses the individual, relationship, community, and societal levels, as shown in Figure 1. Although designed for a different phenomenon, her framework was appropriate for understanding CHPs’ experiences because it clearly accounts for the multilevel contexts that shape their roles in CVD prevention. At the individual level, there are factors related to CHPs as implementers of CVD interventions; at the relationship level, there are factors related to the primary healthcare team and the link or referral to health facilities. At the community level, factors related to community structures and relationships, as well as societal-level policies and structures, influence CHP’s role and resources. This model guided the development of themes, the organisation and interpretation of findings across its four levels, with barriers and facilitators in CVD prevention by CHPs aligned with each level.

### Data analysis

All interviews were audio-recorded and transcribed verbatim. Prolonged immersion in the data was achieved by repeatedly listening to the audio recordings while reviewing the transcripts for accuracy. Interviews conducted in Kiswahili were first transcribed in Kiswahili and then translated into English. The translations were reviewed and refined by the researchers to ensure correct meaning and contextual appropriateness. Both the original Kiswahili transcripts and the English versions were compared and reviewed by a Swahili literacy expert. Final English transcripts were read alongside the original audio recordings. The transcripts were anonymised by sequentially numbering them in the order of the interviews and stored securely prior to analysis.

Data were analysed manually using both inductive and deductive approaches, beginning at the outset of data collection and continuing throughout the data collection period. This was conducted by the first author, with peer review and validation by co-authors. Data analysis was guided by a five-step qualitative content analysis as described by Graneheim and Lundman (32), as shown in Table 1. This involved transcription of audio recordings and repeated reading of transcripts to gain an understanding of the content; identification of meaning units through line-by-line reading; shortening these units to make the data more manageable; and labelling with codes derived directly from the data. Grouping of codes into subcategories, then into categories based on their similarities and differences. And further abstraction of categories into themes that aligned with the four levels of the socioecological model.

**Table 1:** Stages involved in the analysis process.

Stage	Application to the study
1. Transcription and reading	All interviews were audio recorded. Recordings were transcribed verbatim for analysis Kiswahili audios were transcribed in Kiswahili and then translated into English using Google Translate. The translated and original transcripts were reviewed and compared by an expert in Swahili literacy, and any necessary amendments were made Transcripts were read multiple times alongside the audio-recorded interviews to verify accuracy and ensure a comprehensive understanding of the content The key ideas and patterns that reflected CHPs’ experiences in CVD mitigation were identified
2. Identification of meaning units	Meaning units were identified in the text through line-by-line reading
3. Condensation and coding	Meaning units were condensed while preserving their original meaning and were labelled using preliminary codes, which were subsequently reviewed with the co-authors. Code modifications were made as required
4. Aggregation into categories	The inductively identified codes were compared, and those that shared a commonality were grouped into subcategories, which were then aggregated into categories based on differences and similarities The first author, in consultation with co-authors, refined the categories through discussion to ensure consensus on code groupings aligned with the study’s purpose
5. Developing themes	Emerging themes from the categories were deductively aligned with the four levels of the socioecological model The identified themes, categories and subcategories were presented in Table 4. To enhance authenticity, direct quotations from participants were incorporated to support the identified themes

### Trustworthiness and rigour

This study enhanced quality through credibility, dependability, confirmability, and transferability, as described by Lincoln and Guba in 1985 (25). To ensure credibility, data triangulation, member checking, peer debriefing, and verbatim quotations from participants were utilised. Dependability was assured through prolonged engagement with participants, a comprehensive audit trail and the pretesting of interview guides. While transferability was promoted through the provision of thick descriptions, the presentation of findings grounded in the socioecological model was supported by participants’ direct quotations. Furthermore, confirmability was enhanced through reflexivity and expert validation. The first author acknowledged prior knowledge and clinical experience in CVD care, ensured openness during interviews, and documented reflections to minimise bias throughout the research process. Finally, a language expert verified the Kiswahili translations to enhance the objectivity of the findings. Table 2 further illustrates how this criterion was employed.

**Table 2:** Approaches used to enhance trustworthiness and rigour.

Criteria	Approaches	Application to the study
Credibility	Data triangulation	The insights from key informants provided a deeper understanding of CHP experiences
	Member checking	This was achieved during and after interviews by purposefully probing participants to clarify their intended meaning
	Peer debriefing	In collaboration with co-authors, the interpretations were verified, offering diverse viewpoints and helping curb personal bias
	Quotations	Participants’ voices, presented verbatim, were used to support the themes
Dependability	Prolonged engagement	Data were collected after rapport-building, followed by interviews averaging 33 min
	Audit trail	Detailed documentation was prepared, including interview guides, transcripts, field notes, coding decisions and changes, and data analysis procedures
	Pretesting tool	Interview guides were pretested with four participants who met the inclusion criteria
Transferability	Thick descriptions	Detailed descriptions of the study setting, participants, and methods for participant selection, data collection, and data analysis are provided
	Use of a theoretical model	A robust presentation of findings is provided, aligning with the socioecological model and incorporating direct quotes from participants
Confirmability	Reflexivity	Having prior knowledge from the literature review necessitated being open-minded, actively listening and using open-ended questions with minimal interruptions. The first author kept a reflective journal to record reactions and assumptions during interviews
	Expert opinion	
		As a nurse with clinical experience in CVD care and prevention through health education. This experience differed from community-based CVD prevention. I remained actively aware of likely individual bias and preconceptions during data collection, analysis, and interpretation, and documented this in a reflective journal to minimise bias
		The language expert reviewed the translation of Kiswahili transcripts to ensure accuracy and completeness

### Ethical considerations

This study obtained approval from the Departmental Research Committee of the School of Nursing and Midwifery, Aga Khan University. Ethical approval was obtained on 18th September 2024 from the Institutional Scientific Ethics and Research Committee and the National Commission for Science, Technology, and Innovation on 11th October 2024, and a written permit from the Kiambu County Health Committee on 14th November 2024, before the commencement of the study. Written informed consent for participation and audio recording was obtained from all participants and was securely stored in lockable cabinets. They were assured of confidentiality and anonymity, with emphasis on the voluntary nature of participation and the right to withdraw at any time without coercion. However, none chose to withdraw. Confidentiality was maintained by conducting interviews in private rooms, ensuring that only the first author could listen to the interviews or their recordings. Audio recordings were captured using a recorder and were securely stored in a password-protected folder on a personal computer. Participants were assigned numerical codes to ensure anonymity while enabling clear attribution of views during analysis.

## Results

### Participants characteristics

This study included 21 participants: 15 CHPs and 6 key informants (CHAs and the link facility in charge), of whom 12 (57.1%) were female. The participants had a wide range of ages (25–71 years), a mean age of 42.9 years, and a mean work experience of 8.43 years (range: 4–20 years). Those who had attained a tertiary level of education were 52.4% (11). All key informants had attained tertiary education. Geographically, they were distributed across five wards. Table 3 shows the participants’ characteristics.

**Table 3:** Characteristics of participants.

Characteristics	Mean (range)	Frequency (%)
Role		
Community health promoter		15
Key informants (CHA and In-charge link facility)		6
Gender		
Male		9 (42.9%)
Female		12 (57.1%)
Education level		
Secondary		10 (47.6%)
Tertiary		11(52.4%)
Region (ward)		
Kamburu		4
Lari-Kirenga		4
Nyanduma		5
Kinale		4
Kijabe		4
Age	42.9 years (25–70)	
Work experience	8.4 years (4–20)	

Four themes emerged from the analysis based on the four levels of the socioecological model: (1) Individual; (2) Relationship; (3) Community, and (4) Societal levels. Under each level are perceived barriers and facilitators shaping CHPs’ experiences in CVD mitigation in Kiambu, Kenya. The findings identified four categories and eleven subcategories of barriers, as well as five categories and eleven subcategories of facilitators for CHPs in implementing community health interventions to mitigate CVD. Data are presented under each theme category and subcategory (Table 4) with supporting quotes from CHPs and key informants.

**Table 4:** Multilevel barriers and facilitators shaping CHPs’ experiences in CVD prevention.

Themes	Categories	Subcategories
Individual level	Role and personal barriers	Financial burdens of supporting the needy
		Knowledge gaps and training needs
		Role conflict
	Motivation and commitment	Passion and volunteerism
		Provision of financial incentives
Relationship level	Systemic and health facility barriers	Staffing and operational gaps
		Medication shortages
	Primary health care team support	Consistent supervision
		Teamwork and collaboration
		Mentorship and technical support
Community level	Community-related barriers	Resistance to CHP
		Low awareness and perception of risk
		Noncompliance with care
	Community support and trust	Leadership support
		Trust and acceptance
Societal level	Insufficient resources	Inadequate financial support
		Inconsistent functionality of equipment
		Lack of protective wear
	Resource support	Capacity building through training
		Provision of equipment
	Policy and structural support	Functional referral system and feedback
		CHP role integration

### Theme 1: individual level

At the individual level, CHPs’ financial and knowledge limitations constrained performance, though intrinsic and extrinsic motivation sustained engagement. The theme yielded two categories and five subcategories.

#### Category 1.1: CHP role and personal barriers

The challenges include the economic burden of supporting vulnerable clients and the strain of balancing high workloads with other responsibilities.

- Subcategory 1. Financial Burdens of Supporting the Needy; CHPs reported using individual income to support patients who lacked money for medications or transportation, while also encountering unmet community expectations for financial or material assistance. Participant 1complained, *“…they will expect you, as a CHP, to provide them with the money or transportation to go to the facility. And you see, I also have my own challenges…” P1.* Community demands were expressed by participant 14, *“… they ask, what did you bring me? ‘They say I told them to eat this, and I have not brought them anything” P14.* Key informant 3 commented, *“… there are times that you will find that they (CHPs) have entered their pockets and given the client money from the little money he has” KI3.*
- Subcategory 2. Role Conflict: Some CHPs serve more than 100 households while others face conflicting obligations as they pursue additional work to increase their income, resulting in an emotional toll from unmet needs. In seeking balance for his roles, Participant 7 said, “*…you have your own work that you are supposed to do. Like I am a farmer, I must attend to my cows. You cannot even divide yourself well. I feel drained. Yes. I am tired. I am drained,”* P7. Key informant 5 explained the difficult choices that CHPs must make. *“… it is difficult for some CHPs to balance their roles as they have their own families, now they have a part of CHPs, so you find that some CHPs are resigning. It becomes difficult to balance the many households, most of which have more than a hundred households*” (KI5).
- Subcategory 3. Knowledge Gaps and Training Needs: CHPs lack adequate knowledge and training on CVD. Participant 9 said, *“My knowledge about these diseases is not very high… I would like to be trained on these diseases that I have researched on Google” (P9).* Key informant 1 confirmed CHPs’ limited CVD knowledge, “*…though their knowledge they are having is not adequate, they need several trainings” (KI1).*

#### Category 1.2: CHP motivation and commitment

Motivators such as volunteerism and financial incentives propel and sustain the CHP role despite obstacles.

- Subcategory 1 Passion and volunteerism: An intrinsic drive, personal commitment, and dedication, even with low or delayed financial compensation, motivated CHPs. Participant 14 pointed out passion for community work, “…*if you are driven by the passion to make sure that the community are healthy, that will make you feel better when things are going the way that you need them to go”* P14. Key informant 3 agreed with passion rather than financial incentives, *“*… *they do their work because of passion. It is not because they were called to come, nor is it because of money*… *it is something they love doing” KI3.*
- Subcategory 2. Provision of Financial Incentives: CHPs received monthly stipends from the county and the government, which enhanced their morale and supported their work. Participant 6 disclosed, *“…they are paying a stipend. Although it is not something we can be proud of, it is just intervening as a kind of motivation,” P6.* Key informant 3 agreed that CHPs receive financial incentives, *“See the stipend the county and the national government give them; it has also motivated them” KI5.*

### Theme 2: relationship level

This level depicts both supportive factors and obstacles experienced by CHPs in preventing and managing cardiovascular diseases within their communities, such as collaboration with primary health care staff and operational challenges affecting referrals of care. Two categories and five subcategories were identified.

#### Category 2.1: systemic and health facility barriers

Structural and operational limitations of medications and staff within the health system impede CHPs’ interventions.

- Subcategory 1. Medication Shortages: The constant unavailability of antihypertensives and antidiabetic medicines at the link facilities, as reported by participant 1, *“The doctor you want, they are not there… the people get disappointed…the drugs, they are never there. You either get one, go for the other one, and buy in a chemist “(P1).* Key informant 5 commented on the withdrawal of CVD risk medications from link facilities, *“…even after referral to the facility, sometimes there are no drugs. We used to have these drugs for hypertension and diabetes. They were all moved to level four.” (KI5).*
- Subcategory 2. Staffing and Operational Gaps; Participants described the limited availability of staff and inadequate responsiveness of health facilities, resulting in the community’s noncompliance with referred care. As echoed by participant 1, *“…when I went last month, the doctors were not there, they said they are overworked… So it has been closed for 2 months now (P1).* Participant 3 explained preference for private facilities, *“…I do not even want to go, I would rather find money to go to a private hospital and get treated. Yes, they should not turn away the patients or speak to them in that harsh way with that bad language “(P3).* Key informant 2 reported difficulties in health facility operations, *“… nothing much is being done there, so the client will end up wasting a whole day in the facility and go back home unattended” (KI1).*

#### Category 2.2: primary health care team support

All participants reported a supportive environment provided by the healthcare team, including CHAs, facility in-charges, and other clinicians that fosters effectiveness.

- Subcategory 1. Consistent Supervision: Supervision by CHAs provided guidance, emotional support, and accountability, as noted by Participant 11, “*CHA, they are very supportive people, if it is a health issue*, *we are supported by CHA*” (P11). Key informant 1 agreed that they offered supportive supervision, “*I have to do some supervision towards the one screening, the same, relating to cardiovascular illnesses*” (KI1).
- Subcategory 2. Teamwork and collaboration: Collaboration with CHAs, focal person and facility staff enabled the allocation of resources, complex task performance, and enhanced patient referrals. Participant 8 noted, “*CHA and Mbugua (the Community focal person), along with these others, are helping me. If you ask them a question, they answer you. If you tell them to come to the villages, they come, and we go with them” (P8).* Participants 10 noted professional support of health workers, *“The doctor in charge comes to the village. When we need him, if we call him, he will come” (P10)*.
- Subcategory 3, Mentorship and Technical Support: Health professionals provided mentorship and advice that strengthened CHPs’ skills and confidence in CVD prevention. Support was noted by participant 7, *“When you have some questions, you just visit the facility and then they give you directions or the directives” (P7).* In affirmative, key informant six explained how they do it, *“After reviewing the client, I take some time to educate them. We do not assume their calls or queries; we guide them. I help them on the referral system” (KI6).*

### Theme 3: community level

This level identifies community support for CHPs and the challenges related to resistance and nonadherence to care that shape CHPs’ role in delivering effective CVD prevention and care in the community. Two categories and five subcategories were identified.

#### Category 3.1: community-related barriers

Participants reported religious, cultural, and economic constraints such as resistance to screening, mistrust of CHPs, low CVD awareness, and poverty, limiting care uptake.

- Subcategory 1. Resistance to CHP Interventions: Participants revealed the root of reluctance and refusal by community members to accept screening, referrals, or CVD care as indicated by participant 9, who said, *“Our family does not believe in medicine, and we do not go to the hospital. And some tell you, do not bring us hospital and medicine stories, we use our local medicines*” (P9). Key informant 2, in agreement, said, *“Those myths that they are not supposed to go to the hospital…those in these churches sometimes prefer not to go to hospitals*” (KI2).
- Subcategory 2. Non-compliance with Care: Participants cited poverty and economic hardship as hindering community members’ access to medications and referral care. Participant 12 lamented that*, “…you have referred the patient, but he does not have the fare to go there”* (P12). The key informant two also noted the challenges, as mentioned, “*I do not have the money to go to the hospital. I do not have that money to go and pay for some medicine. I am not going to go because I do not have the financial support*” (KI2).
- Subcategory 3. Low Awareness and Perception of Risk: The symptomless nature and low awareness of CVD risk factors result in delay, neglect, or refusal of preventive interventions by the community at risk. As shown by participant 3’s remarks. *“*… *they did not know they had hypertension, they just said they had a bad headache because the community members are not very informed, they go and take pain killers*” *(P3).* In agreement, key informant 2 stated, “*…there is denial in the community that you go to a homestead, and they say they are not sick; they do not have those diseases. So, they do not allow you to come and monitor their BPs” (KI2).*

#### Category 3.2: community support and trust

Most participants experience acceptance, trust and support from the communities they serve.

- Subcategory 1. Community Trust and Acceptance: The recognition and reliance by the community on CHPs build a supportive and trusting environment, as reported by participant 7, *“They accepted me as their CHP. They acknowledge me together with the support that I normally give them…and say they are always happy with what we normally do” (P7).* Key informant 3 said, *“They are recognised because of the work that they are doing…the community embraces them, and they can know that this is the person they can run to, in case of an emergency” (KI3).*
- Subcategory 2. Community Leadership Support: Community leaders, including chiefs and assistant chiefs, were involved in mobilisation efforts that expanded community reach, as reported by key informant 5, *“…like the chiefs. They help them mobilise, maybe in the barazas, where they go for those outreaches which enable them to reach many people” (KI5).* CHPs relied on local leaders’ hostility as explained*, “So, in case of hostility, the chief can intervene, the sub-chief can intervene, and then you have access to the household” (P6).*

### Theme 4: societal level

At this level participants receive essential inputs that enable them to carry out their roles in CVD mitigation effectively. At the same time, some supplies are not provided, and remuneration is insufficient to meet CHPs’ needs. Moreover, their role is formally recognised through inclusion within the primary healthcare system and support from multiple sectors. Three categories and seven subcategories were identified.

#### Category 4.1: insufficient resources

Participants reported that some supplies are not provided, and the remuneration is inadequate to meet their needs.

- Subcategory 1. Inadequate financial support: CHPs experience economic strain due to low stipends and payment delays. This was echoed by participant 14 in these sentiments, *“…we are given little money, it is little, and it does not reach us in time.” (P14).* In agreement, key informant 3 said, *“The government is providing a stipend, which I think is a little compared to whatever they do out there.” (KI3).*
- Subcategory 2. Inconsistent Functionality of equipment, including frequent battery depletion and equipment malfunctions in BP machines and glucometers, hinders CVD screening. Participant 13 expressed dissatisfaction due to a lack of funds to replace batteries, *“They are not providing the batteries. We lack money, such as for batteries for the machine, so you cannot measure (BP) for community members” (P13)*. Key informant 5, in agreement, said, *“The BP machine’s batteries are out of stock, and the county takes too long to supply them. Sometimes they must go into their pockets to buy the batteries.” (KI5).*
- Subcategory 3. Lack of protective wear: The absence of basic protective gear in harsh weather hindered participants’ safety and ability to visit homes and conduct outreach. Participant 9 pointed out, *“When it is raining, you want to go help and to visit someone, but you do not have raincoats and umbrellas, we are not provided with them” (P9).* Key informant 3 noted the risks exposed to CHPs, *“It is really raining… the more it rains… the more it is muddy, and flu and colds increase… the more we are putting these CHPs at risk” (KI3).*

#### Category 4.2: resource support

Participants receive essential inputs that enable them to carry out their roles in CVD mitigation effectively.

- Subcategory 1. Capacity building through training: Most participants cited training from the MOH, county, private hospitals, and NGOs that strengthened their knowledge of CVD and service delivery. Participant 5 commented, *“We also have trainings at the community level, brought to us by the county… non-governmental organisations. They come with other trainings” (P5).* This was affirmed by key informant 3, who explained, *“We always have monthly meetings, where we discuss* var*ious topics. We always keep them informed about emerging issues and changes” (KI3).*
- Subcategory 2. Provision of equipment and tools: Participants were provided with screening tools, mainly BP machines and glucometers, in their CHP kit that empower them to detect and monitor CVD risks. Participant 14 said, *“We have a blood pressure machine. We have the blood sugar machine” (P14).* In agreement, key informant 1 pointed out, *“…they were given a BP machine and a glucometer to do screening at the community level” (KI1).*

#### Category 4.3: policy and structural support

Formal systems and frameworks were seen to promote CHPs’ effective interventions.

- Subcategory 1. Functional Referral System and Feedback: Referral and feedback mechanisms improved continuity of care and validated CHPs’ role, as shown by participant 9, *“We have written a referral form for him, if he comes here and they attend to him, they treat him quickly. And they provide you with a report as well” (P9).* In agreement with a working referral, key informant 4 said, *“When they refer the cases from the community to our facility, they do not go through that process of triage; we fast-track those cases (KI4).*
- Subcategory 2. CHP Role Integration in Primary Health Care Systems: Clear responsibilities, referral linkages, and facility support characterised CHPs’ formal recognition as explained by participant 3 *“…they (county) came to trust us or know us as a CHP in my area, so even if something happens, they are the first to ask you or call you or come to you to get that information” (P3).* In agreement with working communication structures, key informant 3 said, *“…there is that communication between them and us, the CHPs have these referral forms they write where the referral is coming from, reason for referral from which unit, from which village, he is from” (KI3).*

## Discussion

This study aimed to explore the barriers and facilitators shaping CHPs’ experiences in preventing CVDs in rural Kenya. By examining CHPs’ and key informants’ perspectives at the individual, relationship, community, and societal levels, the study sought to gain an in-depth understanding of the contextual factors influencing their roles in community-based CVD prevention. The findings reveal a complex interplay of enabling and hindering factors shaping CHPs’ experiences with CVD prevention interventions.

### Barriers faced by CHPs in mitigating CVD in the community

The findings of this study align closely with previous research in sub-Saharan Africa, highlighting similar challenges among community health workers, including some CHPs being responsible for over 100 households, exceeding their allocated caseloads (24). CHPs in Kenya experience a heightened workload due to shortages Mwenda (33) and the addition of CVD tasks to their traditional duties. Engagement in income-generating activities to supplement low earnings further conflicted with CHPs’ performance in CVD prevention. This echoes findings in Uganda and Malawi that multiple responsibilities undermine CVD efforts, intensifying burnout and attrition risks (34, 35).

Although CHPs received compensation, the payments were low and inconsistent, likely affected by changes in government policy, economic conditions, and competing budget priorities (36). Comparable results in Uganda and Nepal by Ndejjo et al. (34) and Tan et al. (37) demonstrate that a lack of financial incentives leads to dissatisfaction and low motivation. Community poverty forces CHPs to use personal funds to cover needs, reflecting deep commitment but eroding morale and income. As noted by Safary et al. (35) in Malawi, shifting costs from health systems to CHPs is unsustainable, highlighting the need for reliable compensation (16).

Training gaps limit CHPs’ self-efficacy in CVD prevention. Similar deficiencies reported in Nepal, Nigeria and Ethiopia hindered accurate screening, diagnosis, and health counselling (37–39). Low knowledge of CVD could be rooted in low levels of formal education. Limited professional development limits up-to-date knowledge on CVD prevention, reducing the accuracy of community health information (40). There is an urgent need to strengthen CHPs’ training in CVD prevention through structured and refresher courses.

At the relationship level, medication unavailability undermined referrals, as untreated patients lost confidence and disengaged from care. Similar drug supply chain gaps have been documented in South Africa and Uganda (18, 34). In Kenya, persistent drug shortages render referral pathways unsuccessful (41). Without consistent access to medications, community confidence and CHP credibility are eroded. Weak referral facilities, marked by understaffing, inconsistent services, and poor responsiveness, undermine care uptake. As noted in South Africa and Rwanda contribute to referral noncompliance (18, 20). Such gaps diminish the impact of CHPs’ screening and referral efforts, thereby weakening community trust.

CHPs experienced community resistance and low uptake of preventive interventions shaped by sociocultural beliefs and misconceptions about CVD. These findings align with religious and cultural hindrances in India and Kenya that delay care (33, 42). On the other hand, low CVD awareness hindered cooperation with CHW mobilisation in Uganda (19) and uptake of referral in urban slums in India (43). These challenges hinder trust and effective implementation of CVD preventive interventions, underscoring the need for cultural competence and sensitivity training for CHPs (40).

Insufficient community knowledge and low risk perception hindered care-seeking, while asymptomatic persons declined interventions. Similar gaps in CVD awareness and perceived risk delay health-seeking and compromise adherence were evident in Rwanda, India, Belgium and England (20, 42, 44). These gaps delay care-seeking and weaken adherence, thereby limiting the impact of early CHP prevention. These perceptions suggest a disconnect between CHPs’ preventive efforts and community perceptions. Non-compliance with linkage care was also common, due to financial constraints. Financial and transportation constraints limited care adherence and uptake in Rwanda, Uganda and Nepal (20, 34, 37). Poverty undermines access to preventive care, and without addressing economic barriers, CVD reduction remains unattainable (40).

At the societal level, lack of protective gear such as raincoats, gumboots, and PPEs affected CHPs ability to conduct follow-ups during the rainy season, resulting in reduced outreach. Comparable shortages of gear were documented in Ethiopia and Nepal (37, 39), where the lack of boots, umbrellas, or torches limited community engagement. In contrast, Ugandan CHWs were provided with protective wear during the rainy season (34). Inadequate provision of basic gear reflects weak system support, undermining service delivery and exposing CHPs to risks. Additionally, operational barriers include malfunctioning BP machines and the frequent need for CHPs to replace batteries. Inadequate equipment in Uganda has also been reported by Ndejjo et al. (19). Financial and logistical barriers limit sustained screening and follow-up care (36, 40).

### Facilitators enabling CHP’S role in mitigating CVD in the community

At the individual level, CHPs demonstrated strong commitment and passion, sustaining service despite limited compensation and deficient resources. This inherent motivation is consistent with findings from Uganda and Malawi (34, 35). This enabled persistent outreach in under-resourced settings. Although volunteerism underpins their role, stipends introduced by the Kenyan government in 2024 alleviated economic strain and reinforced engagement. Such financial support mirrors that in Nepal, where CHWs received modest payments (37), but contrasts with non-financial incentives in Uganda and Ethiopia (34, 39). Regular incentives remain critical for sustaining CHP motivation and validating their role (16).

At the relationship level, CHPs consistently benefited from supervisory support, which provided them with technical guidance and encouragement. Like Ugandan experiences, supportive supervision fosters morale and performance (34). Collaboration with facility staff further reinforced referral structures and strengthened the CHP’s role, unlike reports of poor cooperation in Nigeria (38). Teamwork in this study improved community trust and continuity of care. Mentorship and technical support also refined CHPs’ skills and clarified practises, with findings consistent in Rwanda (20). On the other hand, limited and infrequent training constrained CVD literacy, with most programmes prioritising maternal and infectious diseases rather than non communicable diseases (36). Consistent with focus on trainings on care of infectious diseases in Uganda and Nepal (34, 37).

At the community level, CHPs’ embeddedness and familiarity enhanced trust, credibility, and acceptance of their interventions. Similar trust-building roles have been observed in Uganda and Ethiopia (34, 39). Confidentiality further reinforced trust, addressing concerns raised in South Africa (45). Local leaders supported CHPs by mobilising communities and endorsing their work, strengthening credibility and participation, as reported in South Africa and Ethiopia (18, 39). Additionally, community leaders facilitated limited access to households that were resistant.

CHPs were equipped with screening and referral tools, unlike in Nigeria and Ethiopia, where equipment scarcity hindered screening (38, 39). Yet the absence of weighing scales and tape measures limited risk assessment, reflecting gaps in comprehensive risk screening. Functional referral structures, including feedback mechanisms, foster continuity and motivation, consistent with findings in Rwanda (20). However, recurrent drug shortages and the absence of clinicians weakened referral success, echoing systemic weaknesses noted in South Africa (18). Finally, at the societal level, structured responsibilities, referral tools, and system embedding reinforced their legitimacy and effectiveness, findings consistent with Rwanda, Ethiopia and other LMICs (20, 36, 39). This promotes morale, role clarity, and system-wide performance.

### Strength and limitations

This study contributes to understanding CHPs’ experiences in CVD prevention by using the socio-ecological model, which highlights facilitators and barriers at the individual, relationship, community, and societal levels. Using interviews with CHPs and key informants provided nuanced insights, while triangulation strengthened credibility. However, reliance on self-reported data may have introduced social desirability bias, despite efforts to ensure confidentiality and reflexivity. The qualitative design and limited sample size reduce generalizability; however, purposive sampling across all geographical wards in teh sub county captured diverse experiences. Findings are context-specific and may not be fully representative of other settings.

### Implications for policy and practise

To ensure manageable workloads, the enforcement of the existing Primary Health Care guidelines, which limit workloads to no more than 100 households, is required. Intensification of the implementation of the household social health insurance registration to improve access to health care. To strengthen community-based CVD prevention, it is necessary to structure community engagement with standard guidelines for home visits, screening schedules, and systematic follow-up to enhance engagement. Prioritising regular, sustainable stipends for CHPs, logistical support, and reliable supply chains for essential medications to reduce CVD risk can enhance CHP performance and continuity of care. The implementation of the national training for CHPs on CVD prevention and care modalities will comprehensively support communities in reducing CVD burden. Further research should involve community members in examining CVD prevention, and observational studies should be conducted to examine how CHPs implement CVD prevention interventions.

## Conclusion

Our study provides significant, insightful evidence from low-income rural communities in Kenya on how CHPs face multilevel barriers and facilitators in CVD prevention. Barriers, including limited training, financial constraints, medication shortages, inconsistencies in the provision of essential supplies and staffing gaps, hinder their efforts. However, their dedication, community support and integration into the health system remain vital for maintaining CVD prevention efforts.

## Data Availability

The original contributions presented in the study are included in the article/supplementary material, further inquiries can be directed to the corresponding author.
